# An effective treatment of experimental osteomyelitis using the antimicrobial titanium/silver-containing nHP66 (nano-hydroxyapatite/polyamide-66) nanoscaffold biomaterials

**DOI:** 10.1038/srep39174

**Published:** 2016-12-16

**Authors:** Minpeng Lu, Junyi Liao, Jing Dong, Jun Wu, Hao Qiu, Xin Zhou, Jidong Li, Dianming Jiang, Tong-Chuan He, Zhengxue Quan

**Affiliations:** 1 Department of Orthopaedic Surgery, The Children’s Hospital, Chongqing Medical University, Chongqing 400010, China; 2 Molecular Oncology Laboratory, Department of Orthopaedic Surgery and Rehabilitation Medicine, The University of Chicago Medical Center, Chicago, IL 60637, USA; 3 Department of Orthopaedic Surgery, The First Affiliated Hospital, Chongqing Medical University, Chongqing 400016, China; 4 Department of Orthopaedic Surgery, Yongchuan Hospital of Chongqing Medical University, Chongqing 402160, China; 5 Research Center for Nano-Biomaterials, Analytical and Testing Center, Sichuan University, Chengdu 610064, China

## Abstract

Effective treatment of osteomyelitis remains a formidable clinical challenge. The rapid emergence of multidrug-resistant bacteria has renewed interest in developing antimicrobial biomaterials using antiseptic silver ions to treat osteomyelitis. However, inadequate local retention and severe cytotoxic effects have limited the clinical use of ionic silver for bone grafts. We recently developed novel porous nano-hydroxyapatite/polyamide 66 (nHP66)-based nanoscaffold materials containing varied concentrations of silver ions (Ag^+^) (TA-nHAPA66) and oxidized titanium (TiO_2_), which was added as a second binary element to enhance antibacterial activity and biocompatibility. In this study, we establish a large cohort of rabbit model of experimental osteomyelitis and investigate the *in vivo* antimicrobial and therapeutic effects of TA-nHP66 biomaterials and their *in vivo* silver release kinetics. We find the TA-nHP66 scaffolds exhibit potent antibacterial activities against *E. coli* and *S. aureus*, support cell adhesion and cell proliferation of pre-osteoblasts, and stimulate osteogenic regulator/marker expression. Moreover, the TA2-nHP66 scaffold exerts potent antibacterial/anti-inflammation effects *in vivo* and promotes bone formation at the lesion site of osteomyelitis. We further demonstrate that TA2-nHP66 exhibits excellent biosafety profile without apparent systemic toxicities. Therefore, the TA-nHP66 scaffold biomaterials may be further explored as an effective adjuvant therapy for infected bone defects and/or osteomyelitis debridement.

Osteomyelitis consists of a wide range of inflammatory bone disorders caused by microbial infections or auto-inflammatory processes^1^. As osteomyelitis can occur at different ages and at preferred localizations in the human skeleton, the incidence of osteomyelitis is approximately 1–2% in the United States and is more prevalent in developing countries with mortality rate as high as 2%^2^,^3^. Bacteria responsible for osteomyelitis usually invade bone-forming osteoblasts, leading to pervasive inflammation, necrosis and bone destruction at the sites of infection^4^. As often refractory to treatment and recurrent, osteomyelitis is considered one of the most challenging medical conditions for Orthopaedic surgeons^5^,^6^,^7^. Meanwhile, Orthopaedic devices are the most common surgical devices associated with implant-related infections, and *Staphylococcus aureus (S. aureus*) is the most common causative pathogen in chronic osteomyelitis^8^,^9^. Current treatment strategies for osteomyelitis involve surgical debridement and systemic and/or local antimicrobial therapies, the later can provide high concentrations of antibiotics at the infected site^10^,^11^,^12^. However, effective treatment of chronic osteomyelitis using antimicrobial agents remains a significant clinical challenge^13^,^14^. Furthermore, increasing numbers of osteomyelitis cases are caused by multiple infections or multi-drug resistant bacterial strains such as methicillin-resistant *Staphylococcus aureus* (MRSA), and possess even more formidable clinical challenges^15^,^16^,^17^. Thus, there is an unmet clinical need to develop novel and effective strategies to combat osteomyelitis.

The use of biomaterials to treat osteomyelitis, especially implant-associated osteomyelitis, holds great promise and has been extensively explored^9^. Silver ions are excellent antimicrobial agents and have been used to treat wound infections and to disinfect water^18^,^19^,^20^,^21^,^22^,^23^. Silver was shown to effectively inhibit resistant bacterial strains such as MRSA^24^,^25^ without developing bacterial resistance^26^,^27^. Silver ions were used to treat chronic osteomyelitis with respectable efficacy^28^,^29^,^30^,^31^. However, it was reported that high concentrations of silver ions may lead to severe cytotoxic effects^32^,^33^,^34^,^35^. Several studies indicate that the incorporation of a second chemical may optimize silver-doped materials with better antibacterial activity and acceptable biosafety^36^,^37^,^38^. However, the *in vivo* efficacy and biosafety profiles of such silver-doped biomaterials are lacking. Thus, it’s important to optimize the silver concentrations in these implant scaffold materials.

We previously developed a scaffold material, nano-hydroxyapatite/polyamide-66 composite (nHP66), which exhibits excellent biocompatibility and osteoconductivity and has been approved for clinical bone tissue engineering in China^39^,^40^,^41^,^42^,^43^,^44^,^45^. As titanium (TiO_2_) is also known to exhibit antibacterial activity with excellent biocompatibility^46^,^47^,^48^, we optimized the nHP66 scaffold material by developing the nanosized titanium (TiO_2_) and silver-co-substituted nHP66 scaffold materials (or TA-nHP66)^49^. We found that co-substitution of titanium (TiO_2_)/Ag-containing hydroxyapatite exhibited significant synergistic long-term bactericidal properties *in vitro*^49^,^50^,^51^.

In this study, we establish a large cohort of the rabbit model of experimental osteomyelitis and investigate the *in vivo* antimicrobial activities of the nanosized titanium/silver-co-substituted nHP66 scaffold materials (TA-nHP66) and the *in vivo* silver release kinetics of the scaffold materials. The TA-nHP66 scaffold materials exhibit potent antibacterial activities on *E. coli* and *S. aureus* bacterial cells, support cell proliferation of pre-osteoblastic cells and stimulate the expression of osteogenic regulators and markers. Moreover, the TA2-nHP66 scaffold material exerts potent antibacterial/anti-inflammation effects and promotes bone formation at the lesion site of osteomyelitis. Lastly, we find that the TA2-nHP66 scaffold material exhibits excellent biosafety profile without detectable systemic toxicities. Thus, the TA-nHP66 scaffold biomaterials may be further explored as an effective adjuvant therapy for infected bone defects and/or osteomyelitis debridement.

## Results

### The titanium/silver-containing nHP66 scaffold materials exhibit potent antimicrobial activity *in vitro*

**Figure 1:**
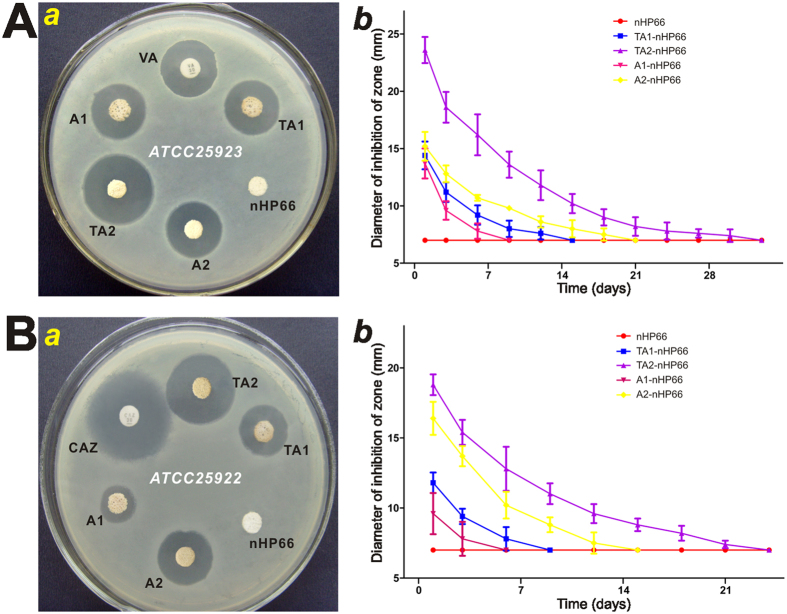
The antibacterial effect of TiO_2_/Ag^+^-containing porous scaffold materials. Antimicrobial activity *in vitro* was evaluated by agar disc-diffusion assay using *S. aureus ATCC25923* (**A**) and *E. coli ATCC25922* (**B**). Uniform discs (1 mm thickness and 7 mm diameter) were placed on to the bacterial inoculated brain heart (BH) agar plates. The plates were incubated under dark conditions for 24 h at 37 °C (a), and the zone of inhibition (ZOI) around the specimen was measured and analyzed (b). The BH agar plates were replaced every 2 days, and ZOI were measured until they disappeared. The vancomycin (VA) and ceftazidime (CAZ) discs (30 μg per tablets) were used as positive controls. Each experiment was repeated five times. Representative results are shown. VA, vancomycin; CAZ, ceftazidime; nHP66, n-HA/PA66 (or nHP66) scaffold material; TA1, 0.22 wt% Ag^+^2.35 wt% TiO_2_-nHA/PA66 (or TA1-nHP66) scaffold material; TA2, 0.64 wt% Ag^+^2.35 wt% TiO_2_-nHA/PA66 (or TA2-nHP66) scaffold material; A1, 0.22 wt% Ag^+^-nHA/PA66 (or A1-nHP66) scaffold material; A2, 0.64 wt% Ag^+^-nHA/PA66 (or A2-nHP66) scaffold material.

The agar disc-diffusion test was used to evaluate the antibacterial effect of the TA-nHP66 scaffold materials against *E. coli ATCC25922* and *S. aureus ATCC25923*. These strains were chosen because *Staphylococcus aureus* and *Escherichia coli* infections account for approximately 75% of clinical osteomyelitis. Based on the analysis of the zone of inhibition (ZOI), the addition of titanium and/r silver rendered the nHP66 scaffold potent antibacterial activities, as compared with antibiotics such as vancomycin (VA) and ceftazidime (CAZ). Specifically, at 24 h after treatment, we found that the ZOI values for nHP66, A1-nHP66, TA1-nHP66, A2-nHP66 and TA2-nHP66 scaffold materials on the *S. aureus ATCC25923* inoculated plates were 7.0 mm, (13.7 ± 1.13) mm, (14.4 ± 1.21) mm, (15.2 ± 1.25) mm, (23.6 ± 1.14) mm, respectively, while the ZOI value of VA to S. aureus was (30.04 ± 2.88) mm (Fig. 1A-a).

Similarly, the ZOI values for nHP66, A1-nHP66, TA1-nHP66, A2-nHP66 and TA2-nHP66 scaffold materials on the *E. coli ATCC25922* inoculated plates were 7.0 mm, (9.6 ± 1.47) mm, (11.8 ± 0.73) mm, (16.4 ± 1.18) mm, (18.8 ± 0.84) mm respectively, whereas the ZOI for CAZ was (17.8 ± 0.85) mm (Fig. 1B-a). These results strongly suggest that the antibacterial activity may be associated with the addition of Ag^+^ and its concentration-dependence in the scaffold materials. The antibacterial activity of TA1-nHP66 was similar to that of A2-nHP66’s, indicating that Ag^+^ and titanium may have synergistic antibacterial effect.

We also determined the changes of the ZOI values of different scaffold materials in both *S. aureus* and *E. coli* changes over time, and found that the maximal ZOI values of the scaffold materials were obtained at 24 h incubation, and then decreased with time (Fig. 1A-b and B-b). Nonetheless, the antibacterial activities as measured by the ZOI of TA2-nHP66 exerted on *S. aureus* and *E. coli* lasted 33 and 12 days, respectively, longer than any other tested scaffold materials (Fig. 1A-b and B-b), indicating the titanium/silver-containing nHP66 scaffold may have the most potent bactericidal effect, compared with the parental nHP66 scaffold and other titanium/silver or silver-containing nHP66 scaffolds.

It’s noteworthy that the potency and duration of antibacterial activities exerted by the scaffold materials seemingly varied among bacterial strains. For example, TA2-nHP66 exhibited more potent and longer duration of antibacterial activities against *S. aureus* cells than that against *E. coli* cells (Fig. 1A-b *vs*. Fig. 1B-b).

### The titanium/silver-containing nHP66 scaffold materials allow efficient cell adhesion and proliferation of pre-osteoblastic cells

**Figure 2:**
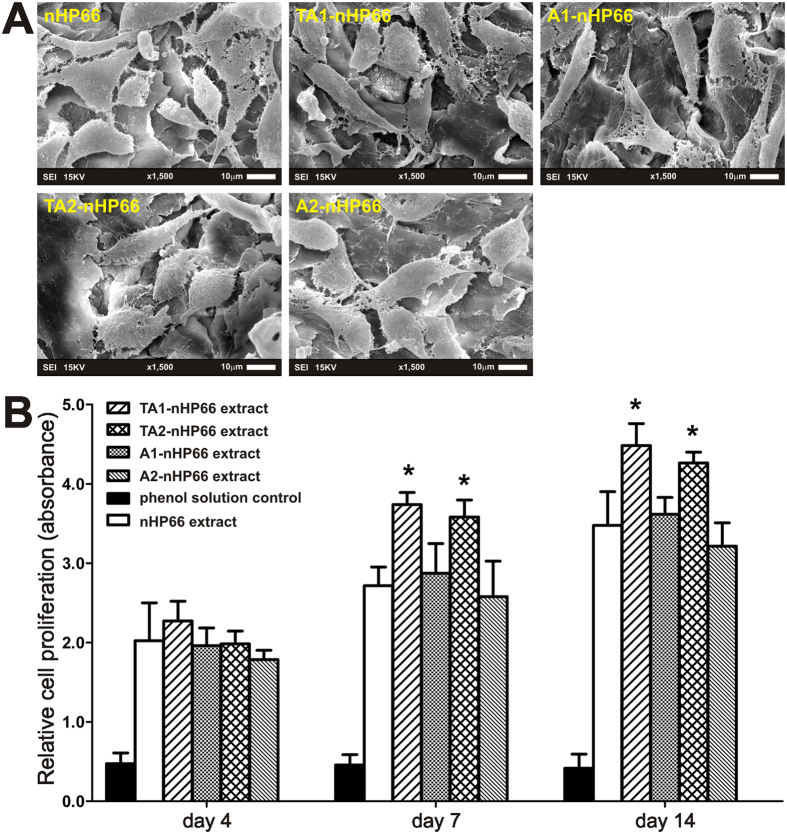
The effect of the TA-nHP66 scaffold materials on the biocompatibility and proliferation of osteoblastic cells. (**A**) SEM micrographs of the MC3T3-E1 cells cultured on different porous scaffold materials for 7 days. (**B**) CCK-8 assay for proliferation of MC3T3-E1 cells cultured with extracts of different porous scaffold materials at 4, 7 and 14 days. “*”p < 0.05.

To test whether osteoblastic progenitor cells are able to effectively attach to the scaffold surface and to facilitate the cell-scaffold interactions, we seeded MC3T3-E1 cells on the scaffold materials and cultured for 7 days. SEM imaging analysis indicated that the MC3T3-E1 cells attached well to the five types of porous scaffold materials with numerous filopodial and pseudopodial extensions (Fig. 2A).

We further analyzed whether the scaffolds would release cytotoxic materials that affect cell survival and proliferation. Using the extracts prepared from different scaffold materials, we assessed the effect of these extracts on cell proliferation of MC3T3-E cells using the Cell Counting Kit-8 (CCK-8) assay. We found that the cell proliferative activities increased in a time-dependent fashion, and the activities were significantly higher in the TA1-nHP66 and TA2-nHP66 groups (p < 0.05) (Fig. 2B). These results demonstrate that the porous TiO_2_-Ag-nHA/PA66 scaffold materials have excellent biocompatibility as they can support cell adhesion and facilitate cell proliferation of MC3T3-E1 osteoblastic progenitor cells.

### The titanium/silver-containing nHP66 scaffold materials stimulate the expression of osteogenic regulators and markers

**Figure 3:**
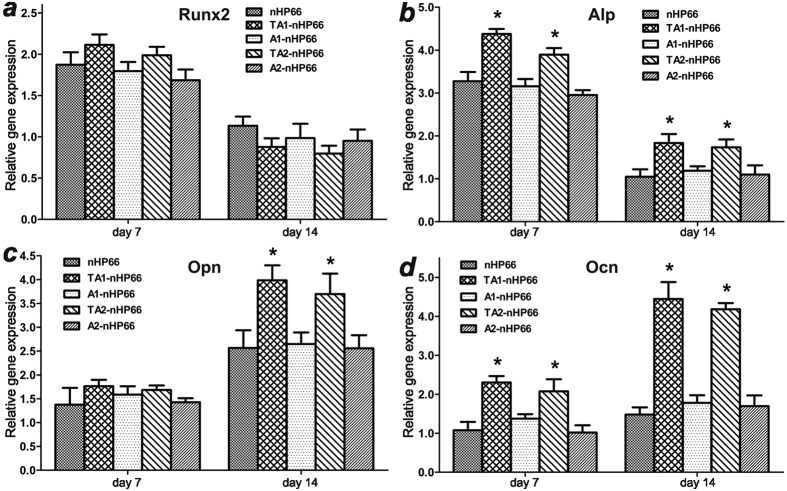
The effect of the TA-nHP66 scaffold materials on the expression of osteogenic regulators and markers. MC3T3-E1 cells were cultured on different porous scaffold materials for 7 and 14 days. RNA was isolated and subjected to qPCR analysis of the expression of *RunX2* (**a**), *Alp* (**b**), *Opn* (**c**) and *Ocn* (**d**) in osteoblast cells. “*”p < 0.05.

To test if the titanium/silver-containing scaffold materials would affect the osteogenic differentiation of pre-osteoblast cells, we cultured MC3T3-E1 cells on the five types of scaffold materials for 7 and 14 days. The expression of osteogenic regulator Runx2 and osteogenic markers Alp, Opn and Ocn was analyzed by qPCR (Fig. 3). Compared with that of the parental nHP66 scaffold material, Runx2 expression was not significantly affected in titanium/silver or silver-containing scaffold groups (Fig. 3a). However, early osteogenic marker Alp and late osteogenic marker Ocn were significantly up-regulated in TA1-nHP66 and TA2-nHP66 groups at both time points (Fig. 3b and d), while another late osteogenic marker Opn was up-regulated in TA1-nHP66 and TA2-nHP66 groups at day 14 time point (Fig. 3c). These results indicate that titanium/silver-containing nHP66 scaffold materials can effectively promote osteogenic differentiation of pre-osteoblast cells *in vitro*.

### The titanium/silver-containing TA2-nHP66 scaffold material exerts potent antibacterial and anti-inflammation effects in a rabbit model of experimental osteomyelitis

In order to determine whether the titanium-silver containing nHP66 scaffold materials can inhibit bacterial growth and promote bone formation *in vivo*, a rabbit model of experimental osteomyelitis was induced with *S. aureus* injected through the tibial metaphysis. After the disease model was confirmed, the animals were divided into three groups and received three treatments: the control group treated with debridement only, the nHP66 group treated with debridement and nHP66 scaffold implantation, and the TA2-nHP66 group treated with debridement and TA2-nHP66 scaffold implantation. The local and systemic symptoms of osteomyelitis were monitored and analyzed at multiple time points for up to 12 weeks. All rabbits recovered well from surgery while two rabbits (one each from the debridement only group and the nHP66 group) died due to septic complications within 4 weeks after osteomyelitis induction.

**Figure 4:**
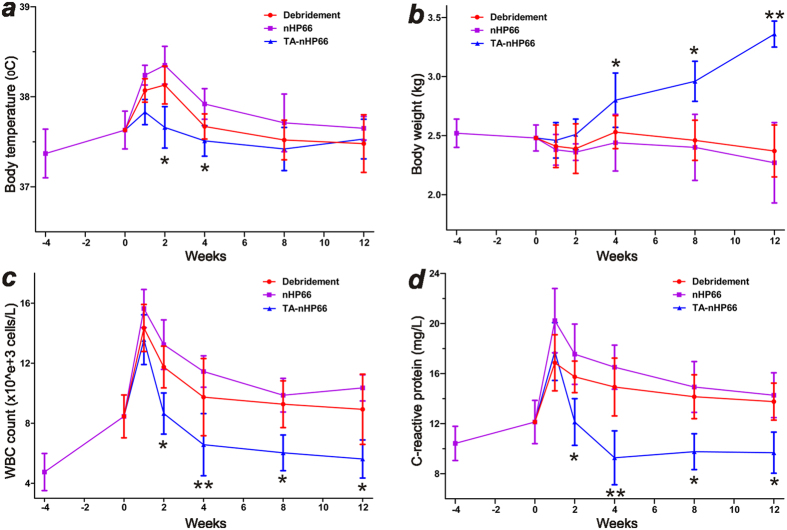
The anti-inflammation features of the TA2-nHP66 scaffold material in the rabbit model of experimental osteomyelitis. Changes in body temperature (**a**), weight (**b**), WBC count (**c**) and C-reactive protein (**d**) were assessed at different time points after the debridement. “*”p < 0.05; “**”p < 0.001 (TA2-nHP66 *vs*. debridement groups).

The average basal body temperature prior to *S. aureus* inoculation was 37.37 ± 0.27 °C. Body temperature slightly increased for all animals before debridement (Fig. 4a), whereas the temperature continued to increase at the first two weeks after debridement in control and nHP66 groups. However, the TA2-nHP66 group exhibited only a slight increase in the first week after implantation, followed by a steady decline to the basal level (Fig. 4a), suggesting the TA2-nHP66 implant may exert potent antibacterial activity, especially compared with the debridement only group (p < 0.05). Nonetheless, the body temperature became stable and maintained close to the basal level, suggesting the animals may have overcome the acute phase of experimental osteomyelitis at 12 weeks after debridement.

Upon the induction of experimental osteomyelitis, the average body weight of the animals slightly decreased to 2.48 ± 0.11 kg (Fig. 4b). After debridement and treatment, the body weight in all three groups showed a noticeable decrease in the first week after treatment, and continued to exhibit a slight decrease in the debridement only group and nHP66 group (Fig. 4b). However, the TA2-nHP66 group, after an initial decrease in the first week, gradually and significantly gained weight, noticeably from 4 to 12 weeks after treatment (Fig. 4b). These results further suggest that TA2-nHP66 implant may exert potent antibacterial activity and that the animals with osteomyelitis may be significantly benefited from TA2-nHP66 implantation.

We also analyzed other inflammation indicators and found the results were in general consistent with the above changes in body temperature and body weight. Compared with preoperative levels, the average white blood cell (WBC) counts slightly increased in all animals after osteomyelitis induction, and the WBC counts of all three groups further increased in the first week after debridement surgery (Fig. 4c). However, the TA2-nHP66 group maintained stable and normal levels of WBC counts 2 weeks after treatment, whereas the WBC counts remained at significantly higher levels in the debridement only and nHP66 groups (p < 0.05) (Fig. 4c). Similar results were found for the serum levels of C-reactive protein (CRP), and the TA2-nHP66 group exhibited reduced and basal levels of CRP at 2 weeks after treatment, while the CRP remained at higher levels in the debridement only and nHP66 groups (p < 0.05) (Fig. 4d).

Taken together, the above results demonstrate that the TA2-nHP66 scaffold material exhibits potent antibacterial activity, compared with debridement only and the parental nHP66 scaffold material.

### The titanium/silver-containing TA2-nHP66 scaffold material inhibits local bacterial infection and promotes bone formation at the lesion site of osteomyelitis

**Figure 5:**
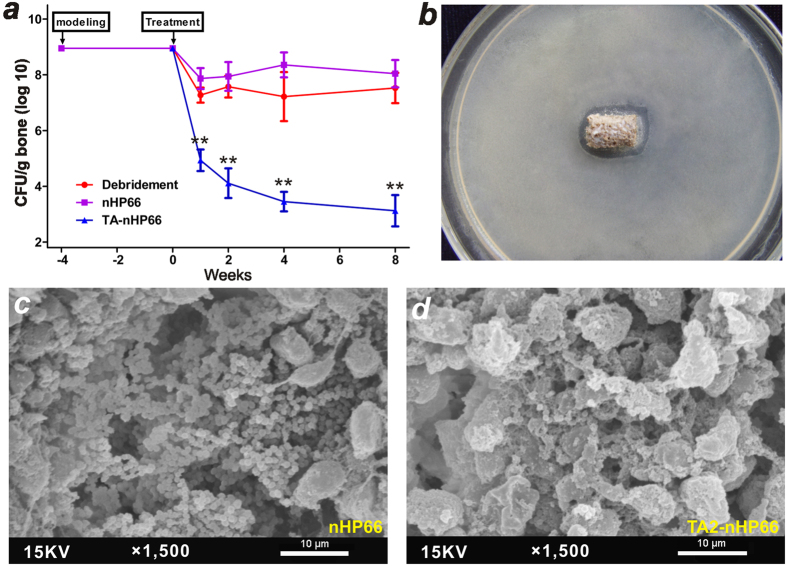
The bactericidal effect of the TA2-nHP66 scaffold biomaterial in experimental osteomyelitis. (**a**) Comparison of the bactericidal effects of three different treatments of the experimental osteomyelitis. “**”p < 0.001. (**b**) Antibacterial effect of the retrieved implant (TA2-nHP66 scaffold material at 8 weeks) on agar plates inoculated with *S. aureus ATCC25923* for 48 h. (**c**,**d**) SEM of the retrieved implants of the control group (nHP66 scaffold, ***c***) and the experimental group (TA2-nHP66 scaffold, **d**), which were removed under aseptic conditions at 8 weeks after debridement. Representative results are shown.

To accurately assess the antibacterial activity of the TA2-nHP66 scaffold material at the implant site, we analyzed the presence of bacterial cells (in terms of colony-forming units, CFUs) by culturing bone samples along with the retrieved implants from the sacrificed animals at each time points. We found that the average CFU values (normalized by grams of bone tissue) at one week after debridement were (1.18 ± 1.04) × 10^5 ^CFU/g, (1.62 ± 1.30) × 10^8 ^CFU/g and (2.24 ± 1.70) × 10^7 ^CFU/g for the TA2-nHP66 group, the nHP66 group, and the debridement only group, respectively (Fig. 5a). The average CFU/g values gradually decreased to (2.32 ± 2.27)  × 10^3 ^CFU/g in the TA2-nHP66 group at week 8, whereas the average CFU/g values of nHP66 and debridement only groups remained at or close to the first postoperative week’s levels (Fig. 5a). The difference in the average CFU values between the TA2-nHP66 group and other two groups was highly significant at all time points (p < 0.001), while there was no statistically difference between the nHP66 group and the debridement only group up to 8 weeks after the debridement surgery (Fig. 5a).

The retrieved TA2-nHP66 scaffold material from the animals at 8 weeks post implantation was shown to retain antibacterial activity, based on the agar disc-diffusion assays (Fig. 5b), while no such activity was observed with the retrieved nHP66 scaffold material at the same time point (data not shown). We further carried out SEM analysis of the bone tissues along with the scaffold implants retrieved at 8 weeks post debridement surgery. We found that significantly fewer numbers of bacterial cells were seen on the surface or in the micropores of the TA2-nHP66 scaffold material while the surface of the nHP66 scaffold was covered with countless bacterial cells (Fig. 5c *vs*. d). Taken together, the above results demonstrate that the TA2-nHP66 scaffold material can effectively control the local infection of osteomyelitis, compared with debridement only and the parental nHP66 scaffold material.

**Figure 6:**
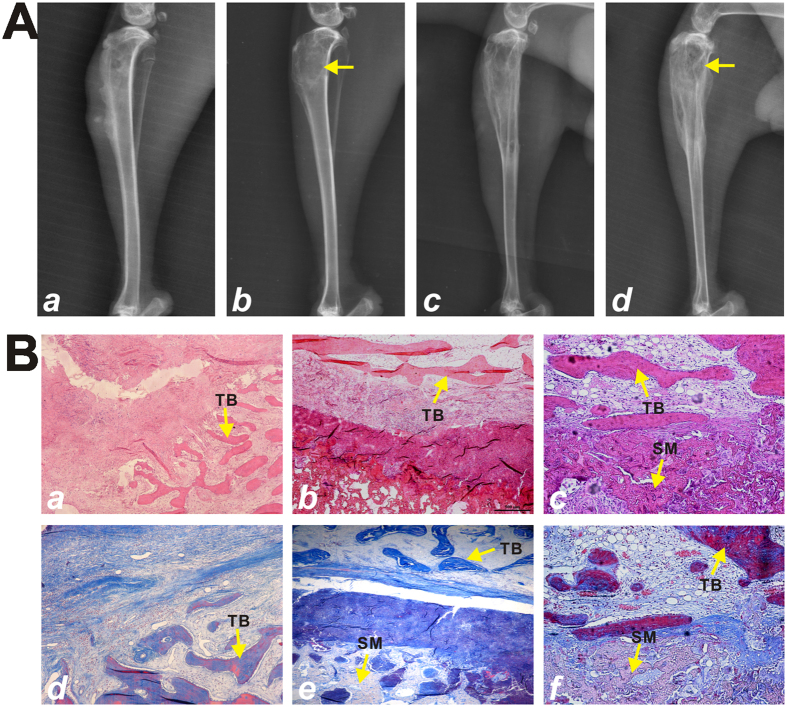
Radiographic and histological evidence of the bactericidal effect of the TA2-nHP66 scaffold material in experimental osteomyelitis. (**A**) Radiographic imaging of experimental osteomyelitis in the course of TA2-nHP66 treatment. (a) Four weeks after the induction of osteomyelitis; (b) After treatment with porous TA2-nHP66 scaffold for 12 weeks (indicated by the arrow); (c) After treatment only with debridement for 12 weeks; (d) After treatment with control nHP66 scaffold for 12 weeks (indicated by the arrow). (**B**) H & E and Masson trichrome staining of bone-Scaffold interface. The implants were retrieved at 12 weeks and subjected to H & E staining (a–c) and Masson trichrome staining (d–f). (a & d) treatment only with debridement; (b & e) treatment with control nHP66 scaffold material; and (c & f) treatment with TA2-nHP66 scaffold material. Representative results are shown. SM, scaffold material; TB, trabecular bone.

To assess the therapeutic effect of the TA-nHP66 scaffold on experimental osteomyelitis, we analyzed the radiographic presentations of the animals sacrificed at 12 weeks after debridement surgery. Radiographic analysis showed the successful induction of osteomyelitis at right proximal tibia of all rabbits with the presence of soft tissue swelling, reduced bone density, bone destruction, and sequestrum bone formation (Fig. 6A-a). At 12 weeks post the debridement surgery, the TA2-nHP66 group showed the formation of new trabecular bone that was well connected to surrounding bone, and the affected proximal tibia almost restored to its normal anatomical structure (Fig. 6A-b). However, soft tissue swelling, osteolytic lesion and reactive periosteal new bone formation were observed in the nHP66 group and debridement only group at the same time point (Fig. 6A-cd).

Histologic analysis further confirmed the radiographic findings on the retrieved bone/implant samples. H & E staining revealed that the debridement only group displayed pronounced inflammation infiltration, bone necrosis and fibrous hyperplasia at the bone lesion site (Fig. 6B-a and B-d), some of which were slightly improved in the nHP66 group (Fig. 6B-b and B-e). However, at 12 weeks after the debridement surgery inflammatory cells were rarely observed in the TA2-nHP66 group, whereas the evidence of new trabecular bone formation and neovascularization was apparent at bone-scaffold interface region (Fig. 6B-c and B-f). Taken together, these *in vivo* results further demonstrate that TA2-nHP66 scaffold material may eradicate bacterial infection locally and repair osteolytic defects caused by osteomyelitis.

### The titanium/silver-containing TA2-nHP66 scaffold material exhibits excellent biosafety profile without detectable systemic toxicities

**Figure 7:**
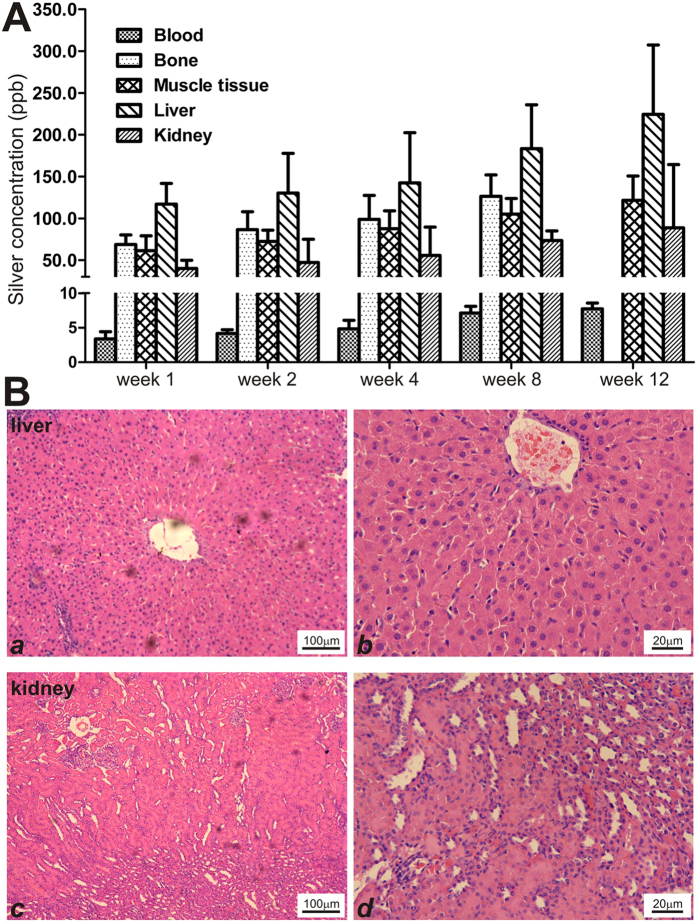
*In vivo* safety profiles of the TA2-nHP66 scaffold implant in a rabbit model of experimental osteomyelitis. (**A**) Silver concentrations of different tissues and organs at different time points of TA2-nHP66 scaffold implantation (after of the debridement surgery). (**B**) H & E histologic evaluation of liver (a & b low and high magnifications) and kidney (c & d low and high magnifications) retrieved from the TA2-nHP66 scaffold implant group at 12 weeks after the debridement surgery.

Towards understanding the *in vivo* toxicological profile of the scaffold materials, we analyzed silver ion release from TA2-nHP66 implant in blood and accumulations in major tissues/organ. We found that the blood silver ion concentrations showed a slight elevation over the 12-week period, but the concentrations were less than 10 ppb (Fig. 7A). The silver concentrations were shown to elevate over time and varied in different tissues (but usually <250 ppb), and the highest concentrations were found in the liver (Fig. 7A). Nonetheless, the silver concentrations in the major tissues were considered low and below the ppm range recommended by the silver safety guideline.

Furthermore, histologic evaluation of the liver and kidney tissues retrieved from the TA2-nHP66 group at 12 weeks after the debridement revealed no significant cytotoxic pathologic findings (Fig. 7B ab *vs*. cd).

Lastly, we analyzed dynamic changes in the clinical panel of liver and kidney serum biomarkers, including ALT (alanine transaminase), AST (aspartate aminotransferase), BUN (blood urea nitrogen), CREA (creatinine) and ALP (alkaline phosphatase) in the TA2-nHP66 group. As shown in Table 1, no significant differences were found for these biomarkers when compared with that of the pre-operative’s (p > 0.05). Thus, these toxicological results demonstrate an acceptable biosafety profile of the titanium-silver containing TA2-nHP66 scaffold material for *in vivo* use.

## Discussion

The effective treatment and management of chronic osteomyelitis remains a formidable clinical challenge for Orthopaedic surgeons^52^. The use of antibiotic-impregnated implant materials, which release antibiotics at local lesions and repair of bone defect caused by debridement, may hold promise as an effective means to treat osteomyelitis^11^,^12^. However, the rapid emerge of antibiotic resistance strains, such as MRSA^15^,^16^, may mandate multidisciplinary approaches to overcoming such challenges, in addition to the development of antibacterial agents with broader antibacterial spectrum.

The development and use of antimicrobial biomaterials has gained significant popularities in treating osteomyelitis, particularly for the implant-associated osteomyelitis^8^,^9^. Silver ions have a long history of being used as antimicrobial agents^18^,^19^,^20^,^21^,^22^,^23^. Historically, silver irons were reported to treat chronic osteomyelitis and infected non-unions^30^,^31^,^53^. More importantly, silver ions were shown to overcome antibiotic resistance in methicillin-resistant Staphylococcus epidermidis (MRSE), MRSA and vancomycin-resistant strains^24^,^54^,^55^. Furthermore, silver was shown to be active against fungi and viral pathogens^25^,^56^.

Although not completely understood, the antimicrobial mechanism of silver irons is generally considered through their binding with microbial DNA, and thus interfering with microbial DNA replication^25^,^57^. Silver irons may also bind to bacterial membrane and/or of bacterial or enzymatic sulfhydryl, amino, imidazole groups of bacterial enzymatic proteins, leading to protein denaturation^57^,^58^,^59^.

While it was reported that bacterial cells are sensitive to silver ions and the antibacterial concentrations of silver irons are as low as 35 ppb^25^, it is known that silver toxicity is dose-dependent and high concentrations of silver ions can inhibit osteoconductivity and osteoblast adhesion, delay wound healing, and exert severe cytotoxicity on a variety of cell types^32^,^33^,^34^,^35^,^60^,^61^.

To minimize the possible silver-related toxicity and to maximize the antimicrobial activity of the silver-doped biomaterials, we developed novel porous TiO_2_/Ag-nHA/PA66 antibacterial nanoscaffold materials (TA-nHP66) using a thermal spraying technique, in which we found that co-substitution of titanium (TiO_2_)/Ag-containing hydroxyapatite exhibited significant synergistic long-term bactericidal properties *in vitro*^49^,^50^,^51^. Our observed synergistic bactericidal properties between silver and titanium were also confirmed by several recent studies^62^,^63^,^64^. However, these studies including our previous ones mostly focused on *in vitro* bactericidal effects, not on *in vivo* disease models. Moreover, the *in vivo* release kinetics and biosafety profiles of silver-doped scaffold materials were not thoroughly studied.

In this study, we established a large cohort of rabbit experimental osteomyelitis and demonstrate that the silver/titanium-containing nHP66 antibacterial scaffold materials exhibit synergistic bactericidal properties. The antibacterial activity of TA-nHP66 biomaterials is dose-dependent of Ag^+^ concentrations. We demonstrate that porous TA2-nHP66 scaffold material has potent antibacterial activity against *S. aureus in vivo*. Furthermore, the TA2-nHP66 biomaterial was shown to promote osteogenesis and had no apparent cytotoxicity in major organs/tissues.

To the best of our knowledge, our studies represent one of the first to determine the *in vivo* dynamic changes of silver concentrations post silver-doped scaffold implantation in osteomyelitis model. It was reported when blood silver concentrations reach 300 ppb toxic side effects would appear, including argyrosis, leucopenia, and liver and kidney damage^65^,^66^. In general, blood silver levels below 10 ppb were considered normal, and the blood concentration should not exceed 22 ppb^65^. Nonetheless, several studies investigated the cytotoxicity of silver ions in a wide range of concentrations *in vitro*. It was shown the IC_50_ of silver concentration was 721 ppb for L929 mouse fibroblasts and 470 ppb for MC3T3-E1 mouse osteoblasts *in vitro*^25^,^67^. No evidence of silver toxicity was observed in the cultured fibroblasts when silver concentrations were below 1,200 ppb^66^.

Here we found that the silver concentrations in blood were <5 ppb at one week after debridement, then slowly increased and reached ~10 ppb at week 12. Furthermore, we did not observe any apparent histological changes of the retrieved liver and kidney tissues at the endpoint, and all serum biochemical markers for liver and kidney functions were within normal ranges. Furthermore, we analyzed that silver distribution among the major tissues/organs for the duration of the scaffold implantation, and found that silver concentrations in liver, bone, muscle tissue and kidney increased with time. The silver concentration in liver is the highest among the examined tissues at all time points, while significant amounts of silver were observed in bone and muscle tissue. The mean silver concentrations in liver were 224.46 ± 83.04 ppb at week 12, compared with 121.63 ± 28.94 ppb in muscle and 88.57 ± 75.72 ppb in kidney. Nonetheless, the silver concentrations in the major tissues were considered low and below the ppm range recommended by the silver safety guideline.

Nonetheless, we did not include any systemic antibiotic administration after debridement surgery in the current *in vivo* studies. It is conceivable that better overall outcomes may be achieved if the animals are treated with antibiotics in combination with local debridement and TA-nHP66 implantation. Moreover, our overall duration of the *in vivo* studies was limited to 12 weeks. A longer period may be needed to fully assess the *in vivo* toxicity of the scaffold biomaterials. These issues should be addressed in future studies.

In summary, using a large cohort of rabbit model of experimental osteomyelitis, we investigated the *in vivo* antimicrobial activities of the TA-nHP66 scaffold biomaterials and their *in vivo* silver release kinetics. We demonstrated that TA-nHP66 scaffold materials exhibited potent antibacterial activities, supported cell proliferation and stimulated the expression of osteogenic regulators/markers. The TA2-nHP66 scaffold was shown to exert potent antibacterial/anti-inflammation effects and to promote bone formation at the lesion site of osteomyelitis. Furthermore, we showed that the TA2-nHP66 scaffold exhibited an excellent biosafety profile without apparent systemic toxicities. Therefore, the TA-nHP66 scaffold biomaterials may be further explored as an effective adjuvant therapy for infected bone defects and/or osteomyelitis debridement.

## Methods

All methods were performed in accordance with the relevant guidelines and regulations outlined by the journal.

### Cell culture, bacterial strains and chemicals

Pre-osteoblastic MC3T3-E1 cells were obtained from ATCC and maintained at 37 °C in complete α-MEM containing 10% FBS (Hyclone, Logan, UT, USA), 100 units/ml penicillin, and 100 g/ml streptomycin at 37 °C in 5% CO_2_. The bacterial strains E. coli ATCC25922 and S. aureus ATCC25923 were obtained from ATCC. Unless indicated otherwise, all chemicals were purchased from Sigma-Aldrich (St. Louis, MO) or Thermo Fisher Scientific (Waltham, MA).

### Preparation of the TA-nHP66 scaffold materials

The porous TiO_2_-Ag-nHAPA66 (TA-nHP66) scaffold materials were prepared as described^44^,^49^,^50^,^51^. Briefly, the TiO_2_ content in the nHAPA66 was maintained at 2.35 wt% while two different silver concentrations were used: TA1-nHP66 (0.22 wt% Ag^+^2.35 wt% TiO_2_) and TA2-nHP66 (0.64 wt% Ag^+^2.35 wt% TiO_2_). For the control materials, we also prepared the porous scaffold materials containing Ag+ only as described^44^,^49^,^68^, with two different silver concentrations, A1-nHP66 (0.22 wt% Ag^+^-nHAPA66) and A2-nHP66 (0.64 wt% Ag^+^-nHAPA66). The macropore sizes of these scaffold materials ranged from 200 to 500 μm.

### Determination of antimicrobial activity *in vitro*

Antimicrobial activity *in vitro* was evaluated by agar disc-diffusion assay^51^. Two bacterial strains, E. coli ATCC25922 and S. aureus ATCC25923, were used in this study. Experimentally, uniform discs (1 mm thick and 7 mm diameter) of different scaffold materials were prepared. 200 μl of bacterial suspension (1.5 × 10^8 ^CFU/ml) was first spread on brain heart (BH) agar plates, and then scaffold discs were gently placed on the surfaces of agar plates. Antibiotics vancomycin and ceftazidime discs (30 μg antibiotic per tablets) were also used as positive controls. The plates were incubated in the dark for 24 h at 37 °C, and the zone of inhibition (ZOI) around each specimen was measured with a digital caliper. BH agar plates were replaced every 2 days, and the ZOIs were measured until they disappeared. Each assay condition was repeated at least five times.

To determine whether the scaffold materials remains antimicrobial activity at 8 weeks after implantation, the retrieved TA2-nHP66 implants were also subjected to the agar disc-diffusion tests using S. aureus ATCC25923 bacterial cells as described above.

### Scanning electron microscope (SEM) analysis

MC3T3-E1 cells were seeded on different scaffold materials (1 mm thick and 7 mm diameter) with 4.0 × 10^4^ cells and incubated at 37 °C in complete medium for 7 days, the medium was removed, and scaffold materials were rinsed with PBS and processed for SEM analysis (using Hitachi S-3000N2) as described^69^. Furthermore, the retrieved scaffold implants of control group and experimental group were removed under aseptic conditions at 8 weeks after debridement and rinsed with PBS and processed for SEM analysis as described^69^.

### Cell proliferation assay

Cell proliferation of different scaffold materials was analyzed by using cell counting kit-8 assay (CCK-8) as described^70^. To test the biocompatibility of the scaffold materials, the extracts of scaffold materials were prepared according to the guidelines specified in ISO10993-12:2012. The 64 g/L phenol solution was used as a control. Briefly, about 1.0 × 10^4^ MC3T3-E1 cells were seeded in 96-well cell culture plates. At 24 h after seeding, the culture medium was removed and different extracts and phenol solution (200 μl/well) were added. At 4, 7 and 14 days, the CCK-8 solution was added and incubated for 2 h. The absorbance was determined at 450 nm using a microplate reader. Six repeats of each assay condition were performed at each time point.

### Analysis of osteogenic gene expression

At 7 and 14 days after incubation, total RNA from osteoblasts grown on different scaffold materials was isolated using the TRIzoI® reagent according the manufacturer’s protocol. The RT-PCR cDNA products were used for quantitative real-time PCR (qPCR) as described^71^,^72^. The expression of osteogenesis-relate genes, RunX2, Alp, Opn and Ocn were analyzed by using the gene-specific primers (Supplementary Table 1)^73^. Gapdh was used as the reference gene.

### Rabbit model of experimental osteomyelitis

The reported animal studies were carried out by following the guidelines approved by the Institutional Animal Care and Use Committee of Chongqing Medical University, Chongqing, China. Experimental osteomyelitis was induced in the tibial metaphysis of 80 healthy New Zealand white rabbits (male, average body weight, 2.52 ± 0.12 Kg) as described^12^. Briefly, when a rabbit was under general anesthesia, a Kirschner wire (Ф1.5 mm) was inserted into the intramedullary cavity at the tibial metaphysis, followed by injecting 1 ml of 5% sodium morrhuate and 0.1 ml of S. aureus suspension (3 × 10^8 ^CFU/ml). The cavity was closed with bone wax. At 4 weeks after injection, five rabbits were sacrificed to confirm the presence of osteomyelitis according to X-ray and histological examination as described^74^. Meanwhile, bacterial cultures from the lesion bone tissues were also established to confirm the S. aureus infection.

The remaining 75 rabbits were treated by focal debridement, and a cortical bone window (1.0 cm × 0.5 cm) was made at the proximal tibia, and randomly divided into 3 groups (n = 25/group): the control group that was treated with debridement only, nHP66 group that was treated with debridement and nHP66 (1.0 × 0.5 × 0.5 cm) scaffold implantation, and the TA2-nHP66 group that was treated with debridement and TA2-nHP66 (1.0 × 0.5 × 0.5 cm; average weight, 85.53 ± 11.26 mg) scaffold implantation. The wound was closed in two layers. Postoperatively, the activity, eating, and wound healing of all rabbits were examined daily. Relevant inflammation indicators such as body weight, body temperature, white blood cell counts (WBC), and C-reactive protein (CRP) levels were measured at multiple time points preoperatively and postoperatively.

### Microbiological evaluation of the retrieved osteomyelitis bone lesion

Quantitative analysis of bacterial colony-forming units per gram of tibial bone was carried out to evaluate the implants’ antimicrobial activity *in vivo* as reported^75^. At 1, 2, 4, and 8 weeks after debridement, the five rabbits from each group were sacrificed. The muscle tissue (within 3 mm of implantation), bone tissue (within 5 mm of implantation) and the implants were collected under aseptic conditions. Each of the harvested bone samples was cut in halves at mid-sagittal plane; half of which was used for silver concentration analysis and the other half was used to microbiological evaluation. Briefly, the samples were crushed to pulverized bone and the final products were weighed. Sterile PBS was added (at 4:1 ratio, v/w) and vortexed for 5 min, followed by 10-fold serial dilutions of the suspension preparations. Lastly, 0.1 ml of the suspension preparations were plated on to the BH agar plates and incubated at 37 °C for 24 h. Colony forming units were counted as described^75^.

### Radiographic and histological analyses

Radiological imaging was performed before debridement and at 12 weeks after debridement. After imaging at the endpoint, the tibial specimens with implants were collected, fixed in paraformaldehyde, decalcified, and subjected to sectioning. The slides were stained with hematoxylin eosin (H & E) and Masson trichrome^76^,^77^,^78^. Histologic evaluation (H & E staining) of liver and kidney tissues was also carried out for the animals sacrificed at 12 weeks after debridement^79^,^80^.

### Determination of silver concentrations in bone and key organs/tissues

When the animals in the TA2-nHP66 group were sacrificed at 1, 2, 4, 8 and 12 weeks after debridement, silver concentrations were determined in blood, muscle tissue (within 3 mm of implantation site), bone (within 5 mm of implantation site, and half of samples), and liver and kidney tissues. Briefly, the samples were collected at different time points and preserved in 10% formalin. Silver concentrations were determined by atomic absorption spectrometry performed at the Chongqing Minerals and Iron Alloy Laboratory Testing Center, Chongqing, China. Each sample was analyzed in triplicates.

### Serological biomarker analysis

General toxicities to liver and kidney in the TA-nHP66 implant group were assessed by analyzing several key serum parameters in the animals sacrificed at each time point. These assays included alanine transaminase (ALT), aspartate transaminase (AST), blood urea nitrogen (BUN) and creatinine (Cr), which were determined by using the commonly-used kits in clinical diagnostic labs (Sichuan Maccura Biotechnology, Chengdu, China).

### Statistical analysis

All quantitative data were described as mean ± SD. Statistical analysis was performed using SPSS 17.0 software. One way ANOVA was performed to detect statistical significances between groups. A p-value of <0.05 was considered statistically significant.

## Additional Information

**How to cite this article:** Lu, M. *et al*. An effective treatment of experimental osteomyelitis using the antimicrobial titanium/silver-containing nHP66 (nano-hydroxyapatite/polyamide-66) nanoscaffold biomaterials. *Sci. Rep.* **6**, 39174; doi: 10.1038/srep39174 (2016).

**Publisher's note:** Springer Nature remains neutral with regard to jurisdictional claims in published maps and institutional affiliations.

## Supplementary Material

Supplementary Table 1

## Figures and Tables

**Table 1:** Serological biochemical markers in the TA2-nHP66 group.

Biomarkers	Preoperative	Week 1	Week 2	Week 4	Week 8	Week 12
ALT(U/L)	49.40 ± 10.69	55.46 ± 9.79*	53.33 ± 7.35*	51.00 ± 11.11*	48.20 ± 5.63*	47.60 ± 8.79*
AST(U/L)	26.20 ± 4.97	28.57 ± 8.61*	29.37 ± 6.54*	27.00 ± 11.62*	24.60 ± 7.23*	26.00 ± 7.65*
BUN(mmol/L)	6.20 ± 2.49	6.87 ± 3.74*	6.95 ± 4.76*	6.40 ± 2.51*	6.20 ± 4.21*	6.40 ± 3.36*
CREA(μmol/L)	87.80 ± 19.69	93.36 ± 14.74*	90.17 ± 18.53*	82.20 ± 13.88*	93.00 ± 18.97*	88.40 ± 13.01*
ALP(U/L)	17.53 ± 5.67	21.75 ± 8.37*	23.18 ± 6.35*	20.76 ± 11.25*	23.15 ± 5.77*	21.37 ± 7.53*

Serum biochemical markers in the TA2-nHP66 group (M ± S, n = 5).

ALT, alanine transaminase; AST, aspartate aminotransferase; BUN, blood urea nitrogen; CREA, creatinine; ALP, alkaline phosphatase. *p > 0.05.

## References

[b1] Beck-Broichsitter B. E., Smeets R. & Heiland M. Current concepts in pathogenesis of acute and chronic osteomyelitis. Curr Opin Infect Dis 28, 240–245, doi: 10.1097/QCO.0000000000000155 (2015). 25918958

[b2] Uskokovic V. Nanostructured platforms for the sustained and local delivery of antibiotics in the treatment of osteomyelitis. Crit Rev Ther Drug Carrier Syst 32, 1–59 (2015). 25746204 10.1615/critrevtherdrugcarriersyst.2014010920 PMC4406243

[b3] Qureshi A. T. . Antimicrobial biocompatible bioscaffolds for orthopaedic implants. J Tissue Eng Regen Med 8, 386–395, doi: 10.1002/term.1532 (2014). 22700366

[b4] Marriott I., Miller J. R. & Sahraei M. Therapeutic strategies against inflammation and bone loss associated with osteomyelitis. Curr Opin Investig Drugs 8, 887–898 (2007). 17979022

[b5] Rao N., Ziran B. H. & Lipsky B. A. Treating osteomyelitis: antibiotics and surgery. Plast Reconstr Surg 127 Suppl 1, 177S–187S, doi: 10.1097/PRS.0b013e3182001f0f (2011). 21200289

[b6] Sanders J. & Mauffrey C. Long bone osteomyelitis in adults: fundamental concepts and current techniques. Orthopedics 36, 368–375, doi: 10.3928/01477447-20130426-07 (2013). 23672894

[b7] Maffulli N. . The management of osteomyelitis in the adult. Surgeon, doi: 10.1016/j.surge.2015.12.005 (2016). 26805473

[b8] Cunningham R., Cockayne A. & Humphreys H. Clinical and molecular aspects of the pathogenesis of Staphylococcus aureus bone and joint infections. J Med Microbiol 44, 157–164, doi: 10.1099/00222615-44-3-157 (1996). 8636931

[b9] Inzana J. A., Schwarz E. M., Kates S. L. & Awad H. A. Biomaterials approaches to treating implant-associated osteomyelitis. Biomaterials 81, 58–71, doi: 10.1016/j.biomaterials.2015.12.012 (2016). 26724454 PMC4745119

[b10] Folsch C. . Coating with a novel gentamicinpalmitate formulation prevents implant-associated osteomyelitis induced by methicillin-susceptible Staphylococcus aureus in a rat model. Int Orthop 39, 981–988, doi: 10.1007/s00264-014-2582-9 (2015). 25380688

[b11] Cao Z., Jiang D., Yan L. & Wu J. *In vitro* and *in vivo* osteogenic activity of the novel vancomycin-loaded bone-like hydroxyapatite/poly(amino acid) scaffold. J Biomater Appl, doi: 10.1177/0885328215623735 (2015). 26686585

[b12] Zhang X. . Teicoplanin-loaded borate bioactive glass implants for treating chronic bone infection in a rabbit tibia osteomyelitis model. Biomaterials 31, 5865–5874, doi: 10.1016/j.biomaterials.2010.04.005 (2010). 20434766

[b13] Ratnayake K., Davis A. J., Brown L. & Young T. P. Pediatric acute osteomyelitis in the postvaccine, methicillin-resistant Staphylococcus aureus era. Am J Emerg Med 33, 1420–1424, doi: 10.1016/j.ajem.2015.07.011 (2015). 26298052

[b14] Xie Z. . Treatment of osteomyelitis and repair of bone defect by degradable bioactive borate glass releasing vancomycin. J Control Release 139, 118–126, doi: 10.1016/j.jconrel.2009.06.012 (2009). 19545593

[b15] Cornejo P. & Mandell G. A. Bone Scintigraphic Findings in MRSA Osteomyelitis. Clin Nucl Med 41, 153–155, doi: 10.1097/RLU.0000000000001067 (2016). 26571442

[b16] Ashizawa N. . Successful treatment of methicillin-resistant Staphylococcus aureus osteomyelitis with combination therapy using linezolid and rifampicin under therapeutic drug monitoring. J Infect Chemother, doi: 10.1016/j.jiac.2015.11.012 (2015). 26732509

[b17] Prieto-Perez L. . Osteomyelitis: a descriptive study. Clin Orthop Surg 6, 20–25, doi: 10.4055/cios.2014.6.1.20 (2014). 24605185 PMC3942598

[b18] Lansdown A. B. Silver in health care: antimicrobial effects and safety in use. Curr Probl Dermatol 33, 17–34, doi: 10.1159/000093928 (2006). 16766878

[b19] Lansdown A. B. A pharmacological and toxicological profile of silver as an antimicrobial agent in medical devices. Adv Pharmacol Sci 2010, 910686, doi: 10.1155/2010/910686 (2010). 21188244 PMC3003978

[b20] Chernousova S. & Epple M. Silver as antibacterial agent: ion, nanoparticle, and metal. Angew Chem Int Ed Engl 52, 1636–1653, doi: 10.1002/anie.201205923 (2013). 23255416

[b21] Alexander J. W. History of the medical use of silver. Surg Infect (Larchmt) 10, 289–292, doi: 10.1089/sur.2008.9941 (2009). 19566416

[b22] Klasen H. J. A historical review of the use of silver in the treatment of burns. II. Renewed interest for silver. Burns 26, 131–138, doi: 10.1016/S0305-4179(99)00116-3 (2000). 10716355

[b23] Lim P. N., Chang L. & Thian E. S. Development of nanosized silver-substituted apatite for biomedical applications: A review. Nanomedicine 11, 1331–1344, doi: 10.1016/j.nano.2015.03.016 (2015). 25943400

[b24] Rode H., Hanslo D., de Wet P. M., Millar A. J. & Cywes S. Efficacy of mupirocin in methicillin-resistant Staphylococcus aureus burn wound infection. Antimicrob Agents Chemother 33, 1358–1361 (1989). 2508545 10.1128/aac.33.8.1358 PMC172654

[b25] Ando Y. . Calcium phosphate coating containing silver shows high antibacterial activity and low cytotoxicity and inhibits bacterial adhesion. Materials Science & Engineering C-Materials for Biological Applications 30, 175–180, doi: 10.1016/j.msec.2009.09.015 (2010).

[b26] Kunkalekar R. K., Prabhu M. S., Naik M. M. & Salker A. V. Silver-doped manganese dioxide and trioxide nanoparticles inhibit both gram positive and gram negative pathogenic bacteria. Colloids Surf B Biointerfaces 113, 429–434, doi: 10.1016/j.colsurfb.2013.09.036 (2014). 24140741

[b27] Feng Q. L. . A mechanistic study of the antibacterial effect of silver ions on Escherichia coli and Staphylococcus aureus. J Biomed Mater Res 52, 662–668, doi: 10.1002/1097-4636(20001215)52:4<662::Aid-Jbm10>3.0.Co;2-3 (2000). 11033548

[b28] Akiyama T. . Silver oxide-containing hydroxyapatite coating has *in vivo* antibacterial activity in the rat tibia. J Orthop Res 31, 1195–1200, doi: 10.1002/jor.22357 (2013). 23589130

[b29] Yonekura Y. . Osteoconductivity of thermal-sprayed silver-containing hydroxyapatite coating in the rat tibia. J Bone Joint Surg Br 93, 644–649, doi: 10.1302/0301-620X.93B5.25518 (2011). 21511931

[b30] Dueland R., Spadaro J. A. & Rahn B. A. Silver antibacterial bone cement. Comparison with gentamicin in experimental osteomyelitis. Clin Orthop Relat Res, 264–268 (1982). 7105586

[b31] Webster D. A., Spadaro J. A., Becker R. O. & Kramer S. Silver anode treatment of chronic osteomyelitis. Clin Orthop Relat Res, 105–114 (1981). 6975686

[b32] Roy M., Fielding G. A., Beyenal H., Bandyopadhyay A. & Bose S. Mechanical, *in vitro* antimicrobial, and biological properties of plasma-sprayed silver-doped hydroxyapatite coating. ACS Appl Mater Interfaces 4, 1341–1349, doi: 10.1021/am201610q (2012). 22313742 PMC3319099

[b33] Drewa T., Szmytkowska K. & Chaberski M. The short term exposition of AgNO3 on 3T3 mouse fibroblasts cell line. Acta Pol Pharm 64, 175–178 (2007). 17665868

[b34] Sudmann E. . Systemic and Local Silver Accumulation after Total Hip-Replacement Using Silver-Impregnated Bone-Cement. Medical Progress through Technology 20, 179–184 (1994). 7877562

[b35] Marin S. . Applications and toxicity of silver nanoparticles: a recent review. Curr Top Med Chem 15, 1596–1604 (2015). 25877089 10.2174/1568026615666150414142209

[b36] Geng Z. . Strontium incorporation to optimize the antibacterial and biological characteristics of silver-substituted hydroxyapatite coating. Mater Sci Eng C Mater Biol Appl 58, 467–477, doi: 10.1016/j.msec.2015.08.061 (2016). 26478334

[b37] Jin G. D. . Synergistic effects of dual Zn/Ag ion implantation in osteogenic activity and antibacterial ability of titanium. Biomaterials 35, 7699–7713, doi: 10.1016/j.biomaterials.2014.05.074 (2014). 24947228

[b38] Gopi D., Shinyjoy E. & Kavitha L. Synthesis and spectral characterization of silver/magnesium co-substituted hydroxyapatite for biomedical applications. Spectrochim Acta A Mol Biomol Spectrosc 127, 286–291, doi: 10.1016/j.saa.2014.02.057 (2014). 24632237

[b39] Zhang Y. . Evaluation of anterior cervical reconstruction with titanium mesh cages versus nano-hydroxyapatite/polyamide66 cages after 1- or 2-level corpectomy for multilevel cervical spondylotic myelopathy: a retrospective study of 117 patients. PLoS One 9, e96265, doi: 10.1371/journal.pone.0096265 (2014). 24789144 PMC4008500

[b40] Xiong Y. . Analyzing the behavior of a porous nano-hydroxyapatite/polyamide 66 (n-HA/PA66) composite for healing of bone defects. Int J Nanomedicine 9, 485–494, doi: 10.2147/IJN.S52990 (2014). 24531621 PMC3894953

[b41] Yang X. . Comparison of anterior cervical fusion by titanium mesh cage versus nano-hydroxyapatite/polyamide cage following single-level corpectomy. Int Orthop 37, 2421–2427, doi: 10.1007/s00264-013-2101-4 (2013). 24057657 PMC3843220

[b42] Zhao Z. . A hollow cylindrical nano-hydroxyapatite/polyamide composite strut for cervical reconstruction after cervical corpectomy. J Clin Neurosci 19, 536–540, doi: 10.1016/j.jocn.2011.05.043 (2012). 22305868

[b43] Xu Q. . Tissue engineering scaffold material of porous nanohydroxyapatite/polyamide 66. Int J Nanomedicine 5, 331–335 (2010). 20517477 10.2147/ijn.s9869 PMC2875726

[b44] Zhang X. . Morphology, hydrogen-bonding and crystallinity of nano-hydroxyapatite/polyamide 66 biocomposites. Composites Part a-Applied Science and Manufacturing 38, 843–848, doi: 10.1016/j.compositesa.2006.08.002 (2007).

[b45] Zhang L., Li Y. B., Wang X. J., Wei J. & Peng X. L. Studies on the porous scaffold made of the nano-HA/PA66 composite. Journal of Materials Science 40, 107–110 (2005).

[b46] Neoh K. G., Hu X., Zheng D. & Kang E. T. Balancing osteoblast functions and bacterial adhesion on functionalized titanium surfaces. Biomaterials 33, 2813–2822, doi: 10.1016/j.biomaterials.2012.01.018 (2012). 22257725

[b47] Pleskova S. N., Golubeva I. S. & Verevkin Y. K. Bactericidal activity of titanium dioxide ultraviolet-induced films. Mater Sci Eng C Mater Biol Appl 59, 807–817, doi: 10.1016/j.msec.2015.10.021 (2016). 26652436

[b48] Imani R. . Biocompatibility of different nanostructured TiO_2_ scaffolds and their potential for urologic applications. Protoplasma, doi: 10.1007/s00709-015-0896-0 (2015). 26497540

[b49] Lv G. Y. . Preparation and antibacterial activity of silver ions-substituted hydroxyapatite/titania. Eco-Materials Processing & Design Vii 510–511, 78–81 (2006).

[b50] Wu X. . The release properties of silver ions from Ag-nHA/TiO_2_/PA66 antimicrobial composite scaffolds. Biomed Mater 5, 044105, doi: 10.1088/1748-6041/5/4/044105 (2010). 20683127

[b51] Lu M. P. . *In vitro* evaluation of antibacterial activity and cytotoxicity of novel nanocomposite material for bone filling. Materials Research Innovations 15, 24–28, doi: 10.1179/143307511X12922272563662 (2011).

[b52] Oh E. J., Oh S. H., Lee I. S., Kwon O. S. & Lee J. H. Antibiotic-eluting hydrophilized PMMA bone cement with prolonged bactericidal effect for the treatment of osteomyelitis. J Biomater Appl, doi: 10.1177/0885328216629823 (2016). 26847915

[b53] Nand S., Sengar G. K., Nand S., Jain V. K. & Gupta T. D. Dual use of silver for management of chronic bone infections and infected non-unions. J Indian Med Assoc 94, 91–95 (1996). 8810203

[b54] Martinez-Castanon G. A., Nino-Martinez N., Martinez-Gutierrez F., Martinez-Mendoza J. R. & Ruiz F. Synthesis and antibacterial activity of silver nanoparticles with different sizes. Journal of Nanoparticle Research 10, 1343–1348, doi: 10.1007/s11051-008-9428-6 (2008).

[b55] Lok C. N. . Silver nanoparticles: partial oxidation and antibacterial activities. J Biol Inorg Chem 12, 527–534, doi: 10.1007/s00775-007-0208-z (2007). 17353996

[b56] Russell A. D. & Hugo W. B. Antimicrobial activity and action of silver. Prog Med Chem 31, 351–370 (1994). 8029478 10.1016/s0079-6468(08)70024-9

[b57] Mijnendonckx K., Leys N., Mahillon J., Silver S. & Van Houdt R. Antimicrobial silver: uses, toxicity and potential for resistance. Biometals 26, 609–621, doi: 10.1007/s10534-013-9645-z (2013). 23771576

[b58] Gordon O. . Silver coordination polymers for prevention of implant infection: thiol interaction, impact on respiratory chain enzymes, and hydroxyl radical induction. Antimicrob Agents Chemother 54, 4208–4218, doi: 10.1128/AAC.01830-09 (2010). 20660682 PMC2944614

[b59] Jung W. K. . Antibacterial activity and mechanism of action of the silver ion in Staphylococcus aureus and Escherichia coli. Appl Environ Microbiol 74, 2171–2178, doi: 10.1128/AEM.02001-07 (2008). 18245232 PMC2292600

[b60] Pauksch L. . Biocompatibility of silver nanoparticles and silver ions in primary human mesenchymal stem cells and osteoblasts. Acta Biomater 10, 439–449, doi: 10.1016/j.actbio.2013.09.037 (2014). 24095782

[b61] Albers C. E., Hofstetter W., Siebenrock K. A., Landmann R. & Klenke F. M. *In vitro* cytotoxicity of silver nanoparticles on osteoblasts and osteoclasts at antibacterial concentrations. Nanotoxicology 7, 30–36, doi: 10.3109/17435390.2011.626538 (2013). 22013878

[b62] Cheng H., Li Y., Huo K., Gao B. & Xiong W. Long-lasting *in vivo* and *in vitro* antibacterial ability of nanostructured titania coating incorporated with silver nanoparticles. J Biomed Mater Res A 102, 3488–3499, doi: 10.1002/jbm.a.35019 (2014). 24178451

[b63] Azimzadehirani M., Elahifard M., Haghighi S. & Gholami M. Highly efficient hydroxyapatite/TiO_2_ composites covered by silver halides as E. coli disinfectant under visible light and dark media. Photochem Photobiol Sci 12, 1787–1794, doi: 10.1039/c3pp50119a (2013). 23824359

[b64] Yu B., Leung K. M., Guo Q., Lau W. M. & Yang J. Synthesis of Ag-TiO_2_ composite nano thin film for antimicrobial application. Nanotechnology 22, 115603 (2011). 21387845 10.1088/0957-4484/22/11/115603

[b65] de la Riviere A. B., Dossche K. M. E., Birnbaum D. E. & Hacker R. First clinical experience with a mechanical valve with silver coating. Journal of Heart Valve Disease 9, 123–129 (2000). 10678384

[b66] Tweden K. S., Cameron J. D., Razzouk A. J., Holmberg W. R. & Kelly S. J. Biocompatibility of silver-modified polyester for antimicrobial protection of prosthetic valves. Journal of Heart Valve Disease 6, 553–561 (1997). 9330181

[b67] Yamamoto A., Honma R. & Sumita M. Cytotoxicity evaluation of 43 metal salts using murine fibroblasts and osteoblastic cells. J Biomed Mater Res 39, 331–340, doi: 10.1002/(Sici)1097-4636(199802)39:2<331::Aid-Jbm22>3.0.Co;2-E (1998). 9457565

[b68] Fan J. . Study on the development of Ag-nano-hydroxyapatite/polyamide66 porous scaffolds with surface mineralization. Sheng Wu Yi Xue Gong Cheng Xue Za Zhi 29, 1119–1124 (2012). 23469542

[b69] Korkusuz F. . Experimental implant-related osteomyelitis treated by antibiotic-calcium hydroxyapatite ceramic composites. J Bone Joint Surg Br 75, 111–114 (1993). 8380599 10.1302/0301-620X.75B1.8380599

[b70] An S., Gao Y., Ling J., Wei X. & Xiao Y. Calcium ions promote osteogenic differentiation and mineralization of human dental pulp cells: implications for pulp capping materials. J Mater Sci Mater Med 23, 789–795, doi: 10.1007/s10856-011-4531-0 (2012). 22190198

[b71] Zheng Y., Li J., Liu X. & Sun J. Antimicrobial and osteogenic effect of Ag-implanted titanium with a nanostructured surface. Int J Nanomedicine 7, 875–884, doi: 10.2147/IJN.S28450 (2012). 22393287 PMC3289444

[b72] Denduluri S. K. . Immortalized Mouse Achilles Tenocytes Demonstrate Long-Term Proliferative Capacity While Retaining Tenogenic Properties. Tissue Eng Part C Methods 22, 280–289, doi: 10.1089/ten.tec.2015.0244 (2016). 26959762 PMC4782028

[b73] Zhang Q. . TqPCR: A Touchdown qPCR Assay with Significantly Improved Detection Sensitivity and Amplification Efficiency of SYBR Green qPCR. PLoS One 10, e0132666, doi: 10.1371/journal.pone.0132666 (2015). 26172450 PMC4501803

[b74] Norden C. W., Myerowitz R. L. & Keleti E. Experimental Osteomyelitis Due to Staphylococcus-Aureus or Pseudomonas-Aeruginosa - a Radiographic-Pathological Correlative Analysis. British Journal of Experimental Pathology 61, 451–460 (1980). 7426395 PMC2041602

[b75] Shirtliff M. E., Calhoun J. H. & Mader J. T. Experimental osteomyelitis treatment with antibiotic-impregnated hydroxyapatite. Clin Orthop Relat Res, 239–247 (2002). 10.1097/00003086-200208000-00027 12151901

[b76] Ye J. . A thermoresponsive polydiolcitrate-gelatin scaffold and delivery system mediates effective bone formation from BMP9-transduced mesenchymal stem cells. Biomed Mater 11, 025021, doi: 10.1088/1748-6041/11/2/025021 (2016). 27097687

[b77] Yan Z. . A Novel Organ Culture Model of Mouse Intervertebral Disc Tissues. Cells Tissues Organs 201, 38–50, doi: 10.1159/000439268 (2016). 26447649 PMC4710565

[b78] Zhang H. . Canonical Wnt signaling acts synergistically on BMP9-induced osteo/odontoblastic differentiation of stem cells of dental apical papilla (SCAPs). Biomaterials 39, 145–154, doi: 10.1016/j.biomaterials.2014.11.007 (2015). 25468367 PMC4258144

[b79] Li Y. . The Calcium-Binding Protein S100A6 Accelerates Human Osteosarcoma Growth by Promoting Cell Proliferation and Inhibiting Osteogenic Differentiation. Cell Physiol Biochem 37, 2375–2392, doi: 10.1159/000438591 (2015). 26646427

[b80] Deng Y. . Antibiotic monensin synergizes with EGFR inhibitors and oxaliplatin to suppress the proliferation of human ovarian cancer cells. Sci Rep 5, 17523, doi: 10.1038/srep17523 (2015). 26639992 PMC4671000

