# Supplementary material for: An effective treatment of experimental osteomyelitis using the antimicrobial titanium/silver-containing nHP66 (nano-hydroxyapatite/polyamide-66) nanoscaffold biomaterials
Source: Sci Rep. 2016 Dec 16;6:39174. doi: 10.1038/srep39174 (PMC5159876; doi:10.1038/srep39174)
Supplement: Supplementary Table 1 [file srep39174-s1.doc]

**An effective treatment of experimental osteomyelitis using the antimicrobial titanium/silver-containing nHP66 (nano-hydroxyapatite/polyamide-66) nanoscaffold biomaterials**

**Running Title: Bactericidal nanoscaffolds for treating osteomyelitis**

Minpeng Lu^1,2^, Junyi Liao^2,3^, Jing Dong^4^, Jun Wu^1^, Hao Qiu^4^, Xin Zhou^4^, Jidong Li^5^, Dianming Jiang**^3^,** Tong-Chuan He^1,2^*, and Zhengxue Quan^3^*

**^1^** Department of Orthopaedic Surgery, The Children’s Hospital, Chongqing Medical University, Chongqing 400010, China

**^2^** Molecular Oncology Laboratory, Department of Orthopaedic Surgery and Rehabilitation Medicine, The University of Chicago Medical Center, Chicago, IL 60637, USA

^3^ Department of Orthopaedic Surgery, The First Affiliated Hospital, Chongqing Medical University, Chongqing 400016, China

**^4^** Department of Orthopaedic Surgery, The Affiliated Yongchuan Hospital of Chongqing Medical University, Chongqing 402160, China

**^5^** Research Center for Nano-Biomaterials, Analytical and Testing Center, Sichuan University, Chengdu 610064, China

* Corresponding Authors

**CORRESPONDENCES:**

**T.-C. He, MD, PhD**

Molecular Oncology Laboratory

Department of Orthopaedic Surgery and Rehabilitation Medicine

The University of Chicago Medical Center

5841 South Maryland Avenue, MC 3079

Chicago, IL 60637, USA

Tel. (773) 702-7169

Fax (773) 834-4598

E-mail: [tche@uchicago.edu](mailto:tche@uchicago.edu)

**Zhengxue Quan, MD, MSc**

Department of Orthopaedic Surgery

The First Affiliated Hospital

Chongqing Medical University

Chongqing 400016, China

Tel/Fax: 011-86-23-8901 2358

Email: [quanzx18@126.com](mailto:quanzx18@126.com)

| **Supplementary Table 1. List of qPCR Primers** | |
| --- | --- |
| Genes | Primers |
| Runx2 | 5'-AGAAGGCACAGACAGAAGCTTGA-3' |
|  | 5'-AGGAATGCGCCCTAAATCACT-3' |
| Alp | 5'- CGGAAGTGAGGCAGGTAG -3' |
|  | 5'- AGAGCCCACAATGGACAG -3' |
| Opn | 5'-CCGAGGTGATAGCTTGGCTT-3' |
|  | 5'-TCCACGCTTGGTTCATCCAG-3' |
| Ocn | 5'- AAGCAGGAGGGCAATAAGGT-3' |
|  | 5'- TTTGTAGGCGGTCTTCAAGC-3' |
| Gapdh | 5'-GCAAGTTCAACGGCACAG-3' |
|  | 5'-GCCAGTAGACTCCACGACAT-3' |
